# Direct Deoxygenation of Phenol over Fe-Based Bimetallic Surfaces Using On-the-Fly Surrogate Models

**DOI:** 10.1021/acs.jpcc.5c05436

**Published:** 2025-10-13

**Authors:** Isaac Onyango, Qiang Zhu

**Affiliations:** † Department of Mechanical Engineering and Engineering Science, 14727 University of North Carolina at Charlotte, Charlotte, North Carolina 28223, United States; ‡ North Carolina Battery Complexity, Autonomous Vehicle and Electrification (BATT CAVE) Research Center, Charlotte, North Carolina 28223, United States

## Abstract

We present an accelerated nudged elastic band (NEB) study of phenol direct deoxygenation (DDO) on Fe-based bimetallic surfaces using a recently developed Gaussian process regression (GPR) calculator. Our test calculations demonstrate that the GPR calculator achieves up to 3 times speedup compared to conventional density functional theory calculations while maintaining high accuracy, with energy barrier errors below 0.015 eV. Using GPR-NEB, we systematically examine the DDO mechanism on pure Fe(110) and surfaces modified with Co and Ni in both top and subsurface layers. Our results show that subsurface Co and Ni substitutions preserve favorable thermodynamics and kinetics for both C–O bond cleavage and C–H bond formation, comparable to those on the pure Fe(110) surface. In contrast, top-layer substitutions generally increase the C–O bond cleavage barrier, render the step endothermic, and result in significantly higher reverse reaction rates, making DDO unfavorable on these surfaces. This work demonstrates the effectiveness of GRR-accelerated transition state searches for complex surface reactions and provides insights into rational design of bimetallic catalysts for selective deoxygenation.

## Graphical abstract

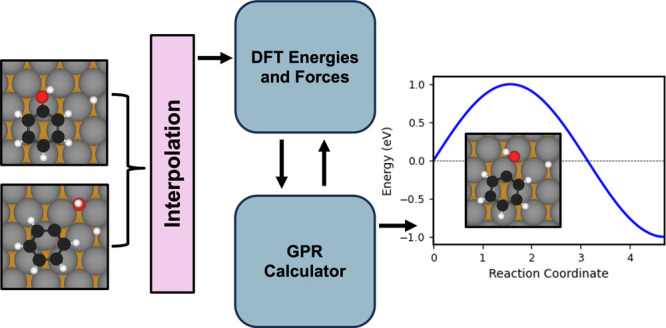

## Introduction

Understanding reaction mechanisms of surface-catalyzed processes is crucial for rational catalyst design. The nudged elastic band (NEB) method is widely used to determine minimum energy pathways (MEPs) and identify energy barriers between local minima configurations.^1–7^ In a practical NEB simulation, the method relies on an elastic band of configurations (images) interpolated between local minima. Each image requires force and energy evaluations from first-principles calculations like density functional theory (DFT) during MEP optimization. Since each electronic structure evaluation typically takes tens or even hundreds of CPU minutes, NEB calculations are computationally very demanding.

In recent years, there have been notable efforts to build machine learning (ML) surrogate models to accelerate NEB calculations.^8–13^ These approaches construct models that closely approximate the true potential energy surface (PES), thereby substantially reducing the number of first-principles calculations required during MEP optimization. In these works, machine learning force fields (MLFFs) were typically trained on a great deal of data in order to achieve the desirable accuracy. If a new configuration cannot be covered by the training data, one has to retrain the MLFFs. In our earlier work, we proposed using a Gaussian process regression (GPR) model to resolve the model update issue for NEB calculations.^14^ This is essentially a hybrid calculator that integrates both DFT and GPR throughout the NEB calculation, and leverages uncertainties for on-the-fly model learning and efficient updates during the NEB optimization allowing it to dynamically adapt to the evolving potential energy surface. This approach is particularly advantageous as it avoids the need for periodic retraining, which can be computationally expensive and may lead to final MEP inaccuracies if not updated frequently enough. Our approach has demonstrated 3–5 times speedup for Pd_4_ cluster diffusion on MgO(100) surface and H_2_S dissociation on transition metal surfaces while maintaining high accuracy.^14^

Motivated by the successes on the cases of small molecules adsorbed on surfaces, we aim to extend this approach to study systems consisting of larger molecules that are generally considered very computationally demanding for routine DFT-based NEB simulations. In this work, we explore the direct deoxygenation (DDO) of phenol on Fe-based bimetallic surfaces, which presents additional challenges due to the structural rearrangements and rotational flexibility associated with bond breaking and formation in such a large molecules on the surface. From a technological perspective, DDO is a particularly attractive hydrodeoxygenation (HDO) pathway, as it yields aromatic products with minimal hydrogen consumption. This offers the potential for HDO under ambient conditions, presenting a promising alternative to traditional HDO processes. However, the DDO of phenolics is challenging due to the high energy barrier associated with the C–O bond cleavage.^15^

Transition metal bimetallic catalysts enhance catalytic activity and selectivity for aromatic production (e.g., benzene, toluene and xylene) compared to monometallic catalysts.^15–22^ Fe-based catalysts demonstrate up to 90% selectivity but suffer from oxidative deactivation. Alloying Fe with other transition metals improves catalyst stability. For example, Hong et al.^23–25^ showed that alloying with noble metals like Pt, Pd, Rh and Ru reduces oxidative deactivation while maintaining high aromatic selectivity, especially in vapor phase conditions.

Most DFT studies of HDO on bimetallic catalysts focus on single-atom alloy (SAA) models that emulate surfaces with low local concentrations of the secondary metal.^26–29^ However, high local coverages of the second metal can significantly influence surface energetics and reaction mechanisms. For example, studies of oxygen reduction reaction (ORR) on Pt_3_Ni­(111) surfaces found that multilayer Pt skin terminations exhibit higher activity.^30,31^ Similarly, Jiang et al.^32^ showed that Co, Fe and Ni terminated surfaces enhance furfural HDO activity compared to pure Pt surfaces, though their DFT calculations were limited to reaction energies without exploring the full minimum energy pathways.

In this work, we focus on Fe-based bimetallic catalysts, specifically Fe(110) surfaces with Co and Ni incorporated via top-layer and subsurface-layer substitution. Co and Ni were selected as they are show potential for enhanced HDO activity and are more earth-abundant compared to noble metals, making them a more sustainable and cost-effective alternative. We employed a GPR calculator to accelerate NEB calculations for exploring the DDO mechanism of phenol on these surfaces. After validating the GPR model on pure Fe(110), where it achieved 3x speedup while maintaining high accuracy, we systematically investigated the bimetallic systems. Our results revealed that subsurface alloying with Co or Ni maintains favorable DDO energetics similar to pure Fe(110), while top-layer alloying generally leads to less favorable reaction pathways.

## Computational Methodology

### Standard DFT-NEB Setup

DFT calculations were performed using the Vienna ab initio simulation package (VASP).^33–35^ Core electrons and electron–electron exchange correlation effects were treated using the projector augmented wave (PAW)^36^ method and optB88-vdW functional,^37^ respectively. We used optB88 functional because van der Waals density functionals (vdW-DF), such as optB88, explicitly include nonlocal correlation term to capture London dispersion interactions.^38,39^ In contrast, semilocal functionals (e.g., PBE, RPBE, SCAN) and hybrid functionals lack this term and therefore cannot be reliably applied to systems, like aromatic molecules adsorbed on surfaces, where dispersion interactions play an important role.^40–43^ Additionally, vdW-DF functionals have been widely used to study aromatic and hydrocarbons on surfaces.^27,29,42,44–46^ Although meta-GGAs combined with vdW corrections (e.g., SCAN + rVV10) may provide higher accuracy, they are more computationally demanding.^41–43^ Spin polarization and dipole correction (applied perpendicular to the surface) were included. Valence electrons were modeled using a plane-wave basis set with a 400 eV cutoff energy. The Methfessel-Paxton smearing method^47^ with 0.1 eV width was used. Calculations were considered converged when energy differences and forces were below 10^–4^ eV and 0.03 eV/Å, respectively. Surfaces were modeled using 4-layer slabs with 15 Å vacuum spacing to prevent interaction between periodic images. The top two layers were allowed to relax while the bottom two were fixed. The Brillouin zone was sampled using a Γ-centered 2 × 2 × 1 Monkhorst–Pack k-point grid.

Transition states (TS) were identified using the climbing image nudged elastic band (CI-NEB) method.^2^ NEB calculations were considered converged when forces on all images were below 0.075 eV/Å, using the FIRE algorithm^48^ implemented in the atomic simulation environment (ASE).^49^ TS structures were verified by calculating vibrational frequencies using central finite differences with a 0.015 Å step size. Within the harmonic approximation, each TS was confirmed by the presence of exactly one imaginary frequency.^50^ For vibrational calculations, the surface was fixed while adsorbates were allowed to relax.

### GPR-NEB Setup

A Gaussian process is a probability distribution over functions that fit a collection of points.^51^ It is characterized by a mean function, typically assumed to be zero, and a covariance function (kernel) that defines the correlation between points. Here the kernel is the radial basis function defined as

$$k\left(x_{i},x_{j}\right)=\sigma_{m}^{2}\exp\left(-\frac{{\left(x_{i}-x_{j}\right)}^{2}}{2l^{2}}\right)$$ 1

where σ_*m*_ and *l* are the hyperparameters that control the magnitude of the covariance function and length scale, respectively. For a set of sample data {***x***, *Y*}, where ***x*** represents the input vector and *Y* is the vector of corresponding observations, the covariance matrix ***C*** is constructed as

$$C_{mn}=C\left(x_{m},x_{n}\right)=k\left(x_{m},x_{n}\right)+\beta \delta_{mn}$$ 2

where β is the noise variance, and **δ**_*mn*_ is the Kronecker delta function. For *N* samples, ***C*** is a square matrix of *N* × *N*. Each sample value can be considered as the linear combination of these covariances.

$$Y_{m}=\sum_{i=1}^{N}G_{\alpha i}C(x_{m},x_{i})$$ 3

Hence, one only needs to determine ***G***_α_ from the previous training data. In matrix form, **Y** = ***C*** ***G***_α_. For a new point ***x***_*N*+1_, the covariance vector ***C***_*N*+1_ is extended as

$$C_{N+1}=\left(\begin{array}{ll} C_{N} & k\\ k^{T} & c \end{array}\right)$$ 4

where *c* = *k*(*x*_*N*+1_, *x*_*N*+1_) + β. And the vector **k** has elements *k*(*x*_*n*_, *x*_*N*+1_) for *n* = 1, ···, *N*. The prediction output and variance for the new point are given by

$$Y_{N+1}=C_{N+1}G_{\alpha }$$ 5

$$\sigma_{N+1}^{2}=c-k^{T}C_{N}^{-1}k$$ 6

The predictive performance of the GPR is improved by optimizing the hyperparameters σ_*m*_, *l*, and β using the maximum likelihood estimation method. For our application, each input structure is represented by a vector of structural descriptors and their partial derivatives. The descriptors encode the local atomic environment and must respect translation, rotation and permutation symmetry. In our implementation, we used the SO(3) descriptors derived from the power-spectrum of spherical harmonic expansion coefficients.^52,53^ Below we summarize the workflow of the NEB calculations with the GPR calculator. For more mathematical and implementation details, see our previous work.^14^

1. Given input initial and final states, an initial trajectory was generated by interpolation between them. And these images together with the initial and final states, along with their DFT-calculated energies and forces served as initial training data for the GPR model.
2. Within each NEB optimization step, the GPR calculator predicts the energy and forces along with the corresponding uncertainties for each image. If the uncertainty in the predictions exceeds a predefined threshold, the model calls the VASP calculator to compute the DFT energy and forces for the NEB calculation and simultaneously adds the simulation results to the training database and then updates the model. This process continues until the NEB calculation converges. Here, we set a threshold for the maximum uncertainty in energy and forces to σ_*E*_ = 0.05 eV/structure for energy and σ_*F*_ = 0.05 eV/Å for forces, respectively.

### Rate Constants Estimation

In addition to MEPs, we estimated the forward rate constants and equilibrium constants using the transition state theory (TST).^54,55^ The forward rate constant *k*_f_ is calculated using the following equation:

$$k_{f}=\frac{k_{B}T}{h}\cdot \frac{Q_{\mathrm{vib}}^{‡}}{Q_{\mathrm{vib}}}\cdot e^{-E_{a}^{‡}/k_{B}T}$$ 7

where *k*_B_ is the Boltzmann constant, *T* is the temperature, *h* is Planck’s constant, *Q*_vib_^‡^ and *Q*_vib_ are the vibrational partition functions of the transition state and reactant state, respectively, and *E*_a_^‡^ is the activation energy barrier. The vibrational partition function is calculated using equation:

$$Q_{\mathrm{vib}}=\prod_{i=1}^{N}\frac{e^{-h\nu_{i}/2k_{B}T}}{1-e^{-h\nu_{i}/k_{B}T}}$$ 8

where ν_*i*_ is the vibrational frequency of the *i*-th mode and *N* is the number of vibrational modes. The reversed rate constant *k*_r_ is calculated using similar equation. The equilibrium constant *K*_eq_ is calculated using the equation:

$$K_{\mathrm{eq}}=\frac{k_{f}}{k_{r}}$$ 9

In this work, both DFT-NEB and GPR-NEB calculations were run in a single compute node of AMD EPYC 9654P 96-Core Processor with 2.40 GHz in our in-house computing cluster.

## Results and Discussion

In this section, we first validate the GPR calculator’s performance by comparing its results against standard DFT calculations for phenol DDO on pure Fe(110) surface. After establishing the accuracy and computational efficiency of our approach, we systematically investigate how surface modification impacts the reaction mechanism by examining both top-layer and sublayer substitution of Fe with Co and Ni. The reaction pathways and energetics are analyzed in detail for each surface configuration. Finally, we compare kinetic parameters across all surfaces to evaluate their catalytic performance and identify promising catalyst designs.

### GPR Calculator Performance on a Pure Fe(110) Surface

We began with test calculations to assess the accuracy and computational efficiency of the GPR calculator by studying the C–O bond scission of phenol on Fe(110) surface. As shown in Figure 1, the MEPs calculated using both the GPR and VASP are nearly identical. The difference in the energy barrier is negligible: 0.813 eV from the GPR compared to 0.827 eV from the pure VASP calculations. Additionally, the TS structures are nearly indistinguishable. In both cases, the OH resides near a bridge site, and the C–O bond is significantly elongated from 1.39 to 1.88 Å for the TS from GPR and 1.96 Å for the TS from VASP. The error in the energy barrier is only 0.014 eV, which is well within the acceptable range for NEB calculations.

**Figure 1:**
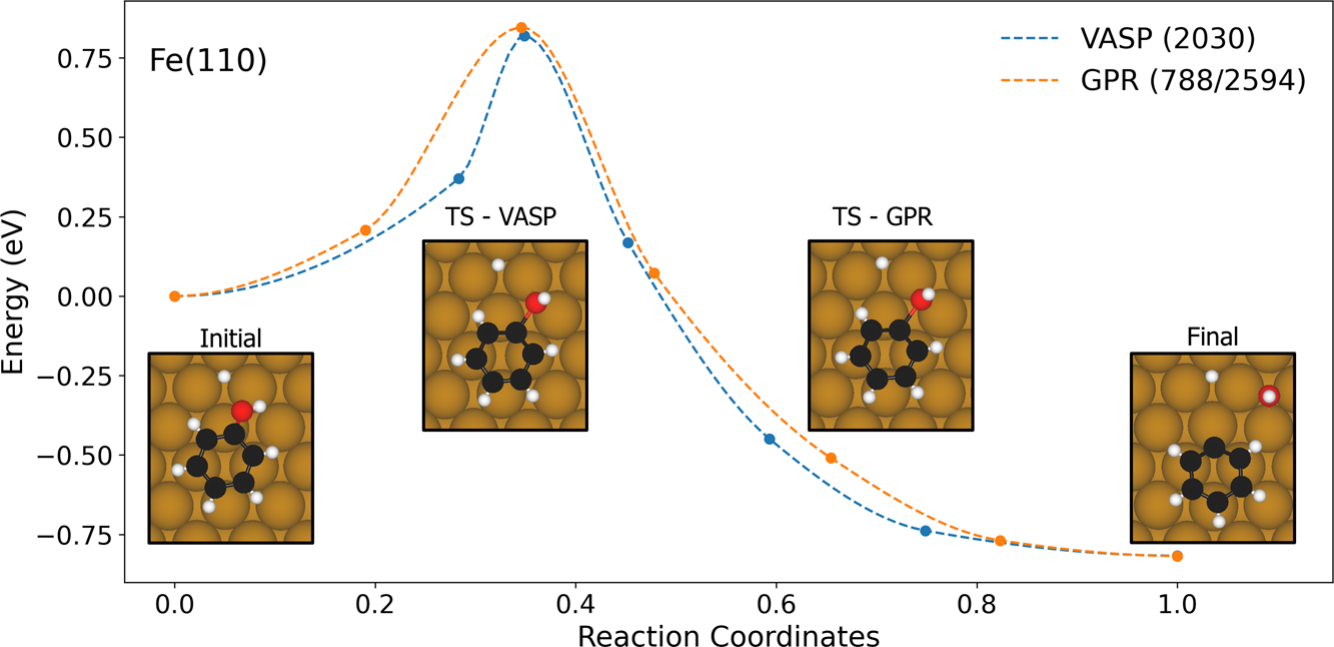
Simulated MEP of deoxygenation of phenol on the Fe(110) surface from both the GPR and pure VASP calculators. The numbers in the parentheses in figure legend are the number of VASP/DFT and GPR calls for the calculators during the NEB calculation. The representative structures along the transition path are also shown in the inset. Brown, white and red spheres represent Fe, O and C atoms, respectively.

In terms of computational efficiency, the GPR calculator completed the MEP optimization in 72 h, requiring 788 DFT calls and 2594 GPR calls. Using the same setup, it costs a total of 216 h based on the VASP calculator with 2030 DFT calls. This demonstrates a 3 times speedup of GPR over the pure VASP-NEB approach.

To understand the acceleration mechanism, we plot Figure 2 to analyze the distribution of GPR and VASP calls during MEP optimization. The first 40 iterations rely predominantly on VASP calculations to build an accurate initial GPR model. Between iterations 40–60, most force and energy evaluations use the GPR calculator, except for the sixth image which requires VASP calculations due to significant structural changes. From iterations 50–210, optimization is dominated by GPR calls, with VASP calculations concentrated near the transition state where major structural rearrangements occur. After iteration 210, the optimization proceeds almost entirely through GPR, requiring only occasional VASP calls for structural corrections. This nonuniform pattern of VASP calls demonstrates how the GPR calculator’s on-the-fly model updates, guided by uncertainty estimates, enable dynamic adaptation to the evolving potential energy surface while maintaining accuracy. As long as the GPR is well trained, it takes only 10–50 s to predict the atomic forces and energies to drive the NEB optimization, while each DFT calculation typically takes 45–55 min for a surface model consisting of 78 atoms in the unit cell.

**Figure 2:**
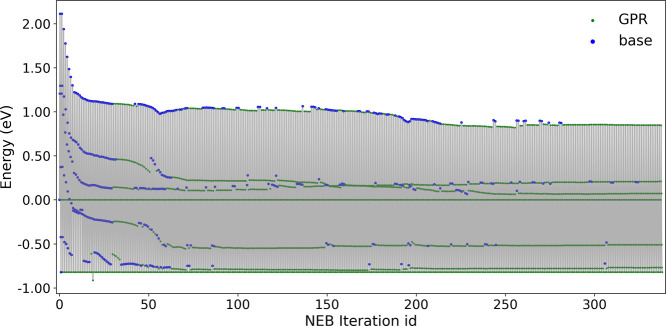
Usage of GPR and base calls during the MEP optimization associated with the GPR-NEB simulation mentioned in Figure 1. In this simulation, each NEB iteration includes 7 consecutive images.

It is important to note that the GPR acceleration efficiency depends on the NEB path quality. In another simulation with a slightly different initial path (see Figure 3), the GPR calculator achieved a more modest 1.8× speedup - completing the MEP optimization in 2.5 days (302 VASP calls, 661 GPR calls) compared to 4.5 days for pure VASP (755 DFT calls). The key difference between these paths lies in the molecular motions during the transition. In the previous case (Figure 1), the TS formation involved both OH group rotation during C–O bond scission and aromatic ring translation/rotation. In contrast, such complex rotational motions were minimal in this example. When present, these additional rotational degrees of freedom can increase the number of iterations needed for NEB convergence. This suggests that optimal performance requires both GPR acceleration and careful selection of the initial reaction path to minimize unnecessary molecular rotations.

**Figure 3:**
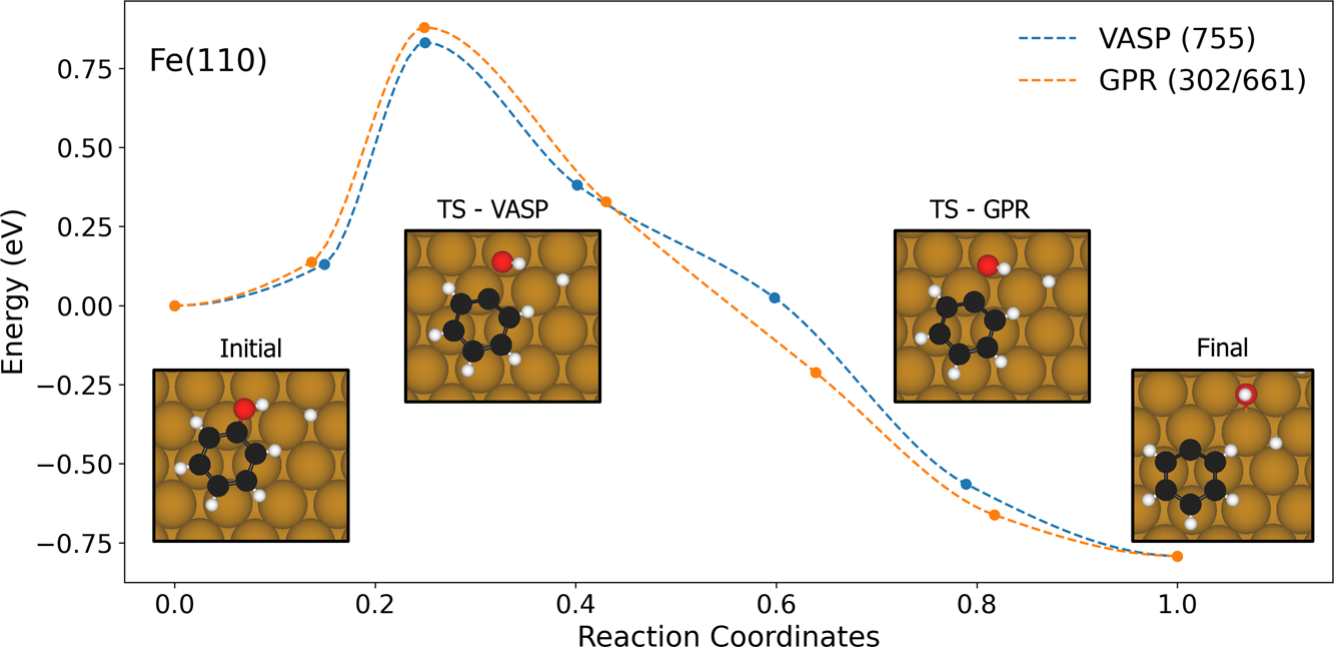
Simulated MEP of deoxygenation of phenol on the Fe(110) surface from both the GPR and pure VASP calculators. The numbers in the parentheses in figure legend are the number of VASP/DFT and GPR calls for the calculators during the NEB calculation. The representative structures along the transition path are also shown in the inset. Brown, white and red spheres represent Fe, O and C atoms, respectively.

### Phenol DDO on Fe-Based Bimetallic Surfaces

Having confirmed the validity of the GPR calculator for studying phenol C–O bond scission on Fe(110), we proceeded to investigate the impact of surface doping on phenol DDO. For a systematic understanding, we considered three types of surface models: (i) pure Fe(110); (ii) bimetallic surfaces with the top Fe(110) layer substituted by Ni or Co; and (iii) bimetallic surfaces with the sublayer substituted by Ni or Co. The reaction mechanism involves two key steps, starting with phenol and hydrogen coadsorbed horizontally on the surface. First, the C–O bond cleaves to form adsorbed phenyl (C_6_H_5_) and hydroxyl species. Subsequently, C–H bond formation occurs as the coadsorbed hydrogen binds to the phenyl group to produce benzene.

For each surface, we studied both the C–O bond cleavage and C–H bond formation steps, resulting in a total of 10 independent NEB simulations. Given that each pure VASP NEB simulation would take 4–9 days, using the GPR calculator allowed us to complete each simulation in just 2–5 days while maintaining accuracy. In the following subsections, we analyze these reaction steps in detail for each surface configuration.

#### Phenol DDO on Pure Fe(110)

The reaction on the pure Fe surface is summarized in Figure 4. The C–O bond cleavage proceeds via a slight translation of the phenol molecule, accompanied by rotation of the OH group to a vertical orientation, with the oxygen atom interacting directly with a bridge site on the surface. At the transition state (TS), the C–OH bond is significantly elongated, increasing from 1.39 to 1.88 Å. During C–H bond formation, the hydrogen atom migrates from a 3-fold hollow site toward a top site at the TS, rising by approximately 0.50 Å above its initial position near the surface. This overall reaction is highly exothermic, with a reaction energy of −1.22 eV. Both the C–O bond cleavage and C–H bond formation steps are exothermic (−0.82 and −0.41 eV, respectively) and exhibit moderate activation barriers of 0.82 and 0.57 eV. These results suggest that DDO readily proceeds on the pure Fe surface, with the C–O cleavage being the rate limiting step.

**Figure 4:**
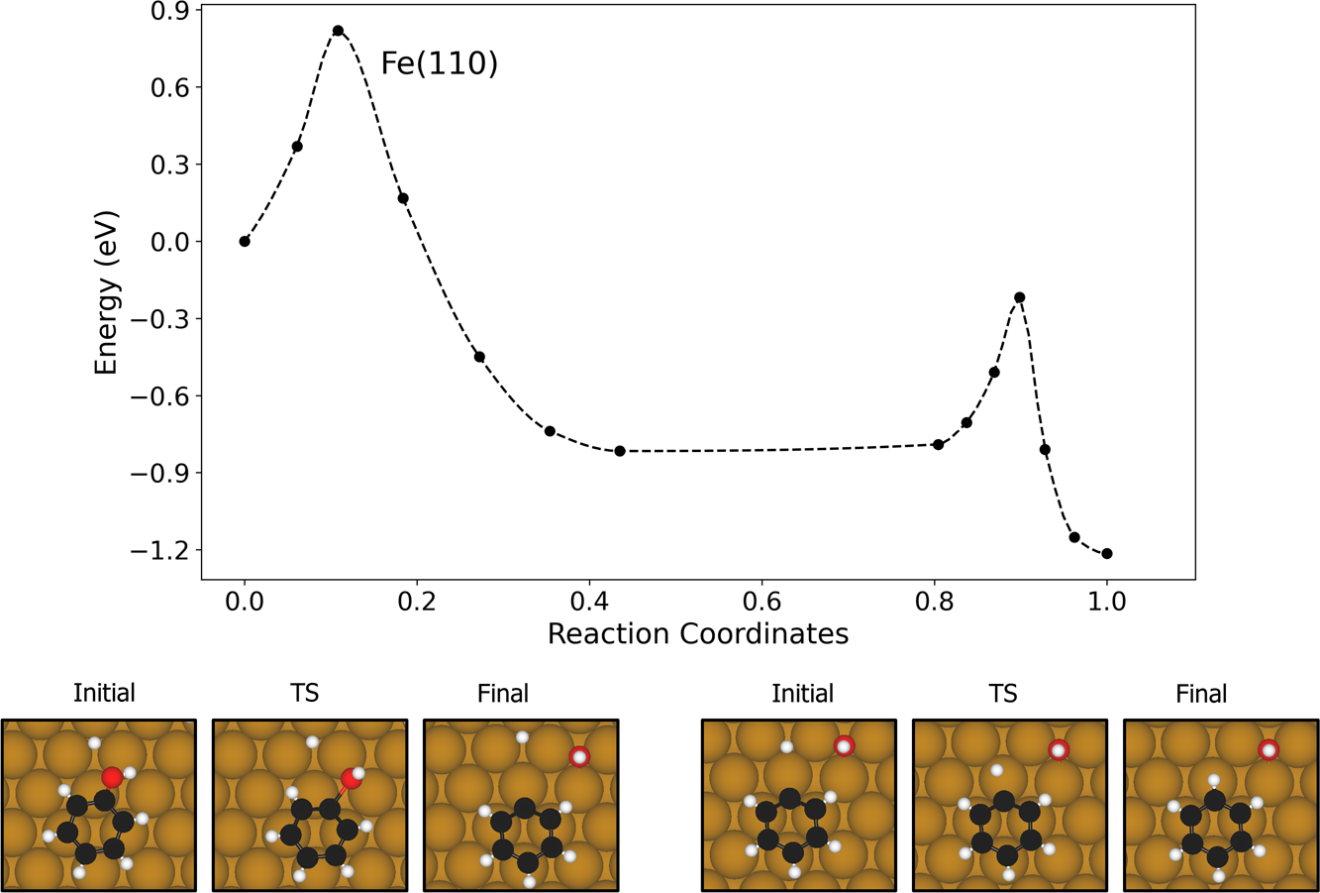
Simulated MEPs of the deoxygenation and hydrogenation steps on the pure Fe(110) surfaces. The initial, transition state (TS) and final structures the elementary steps are shown below the MEPs. Brown, white and red spheres represent Fe, O and C atoms, respectively.

#### Phenol DDO on the Top-Layer Substitution

The MEPs on top-layer substitution surfaces are shown in Figure 5. The C–O cleave proceeds via migration of the OH group, with minimal rotation, toward a top site at the TS. The C–O bond is significantly elongated from about 1.39 to 2.07 Å for Co and 1.38 to 2.21 Å for Ni. The C–H bond formation occurs via a similar mechanism to the pure Fe, with the hydrogen migrating from a 3-fold hollow site to a top site at the TS, rising by approximately 0.77 Å above its initial position near the surface.

**Figure 5:**
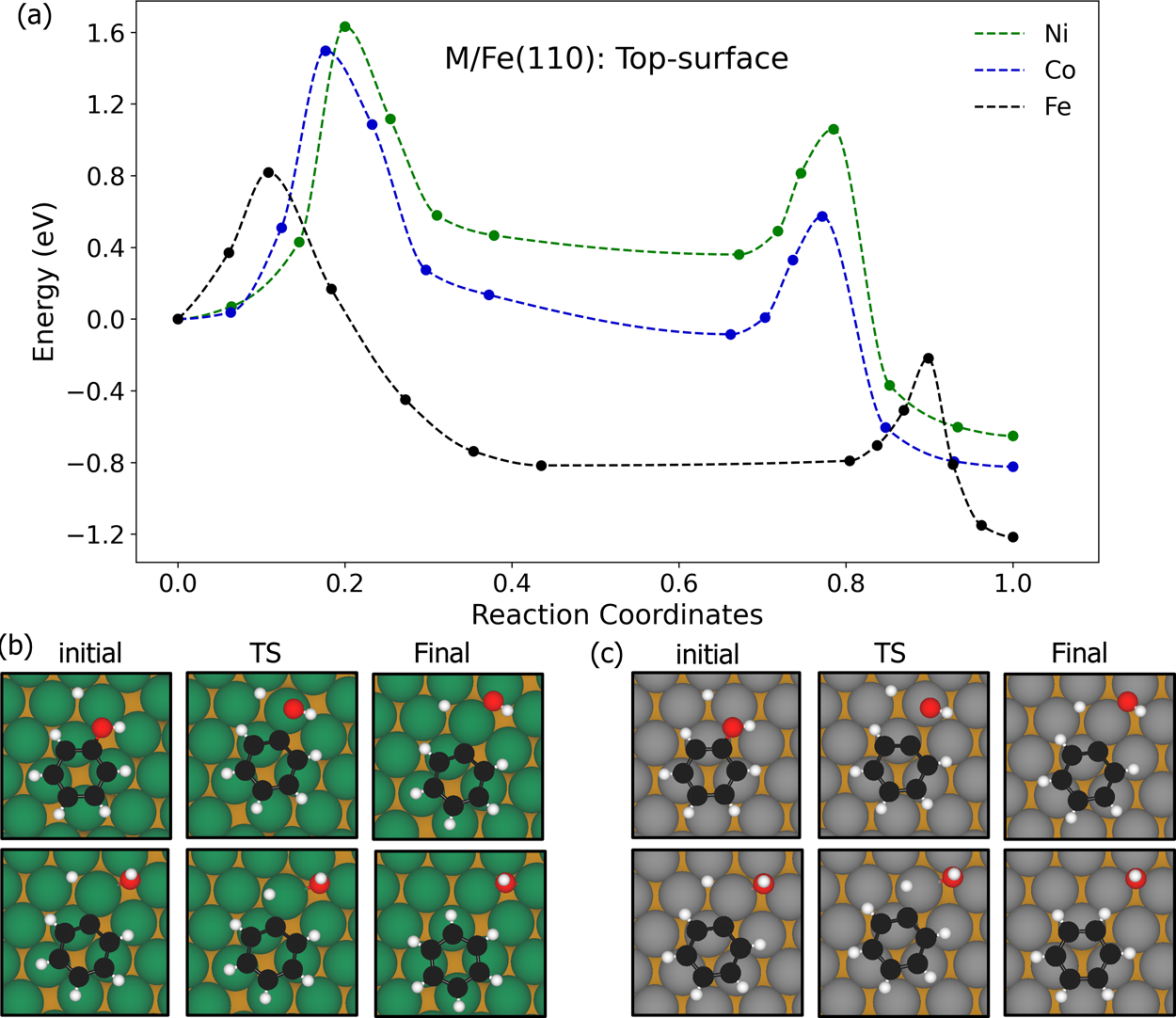
(a) Simulated MEPs of the deoxygenation and hydrogenation steps on Co and Ni top-layer substitution surfaces. (b) Initial, transition state (TS) and final structures for the C–O bond cleavage (top row) and C–H formation (bottom row) steps on the Ni top-surface. (c) Initial, transition state (TS) and final structures for the same steps for the Co top-surface. Brown, green, gray, white, and red spheres represent Fe, Ni, Co, O, and C atoms, respectively.

The overall reaction is moderately exothermic, with the reaction energy of −0.82 eV for Co and −0.65 eV for Ni surfaces. However, the C–O bond cleavage on these surfaces is energetically unfavorable, exhibiting high activation barriers of 1.50 and 1.63 eV, and endothermic reaction energies of 0.14 and 0.47 eV, for Co and Ni surfaces, respectively. In contrast, C–H bond formation is more favorable, lower activation barriers of 0.66 and 0.70 eV, and exothermic reaction energies of −0.74 and −1.01 eV, for Co and Ni surfaces, respectively. These results indicate that C–O bond cleavage is the rate limiting step on these surfaces. Although the overall reaction is exothermic, the unfavorable energetics of the initial cleavage suggest that DDO is not readily facilitated on these surfaces.

#### Phenol DDO on the Sublayer Substitution

Figure 6 summarizes the overall reaction on the sublayer substitution surfaces. Similar to the pure Fe surface, the C–O cleavage also proceeds via a slight translation of the phenol molecule, accompanied by rotation of the OH group to a vertical orientation, with the oxygen atom interacting directly with a bridge site on the surface. TS structures are also similar to that of the pure Fe surface, with the C–O bond elongated from 1.39 to 1.92 Å for Co and 1.38 to 1.88 Å for Ni surfaces. The C–H bond formation also occurs similarly to the pure Fe surface, with the hydrogen atom migrating from a 3-fold hollow site toward a top site at the TS, rising by approximately 0.52–0.79 Å above its initial position near the surface.

**Figure 6:**
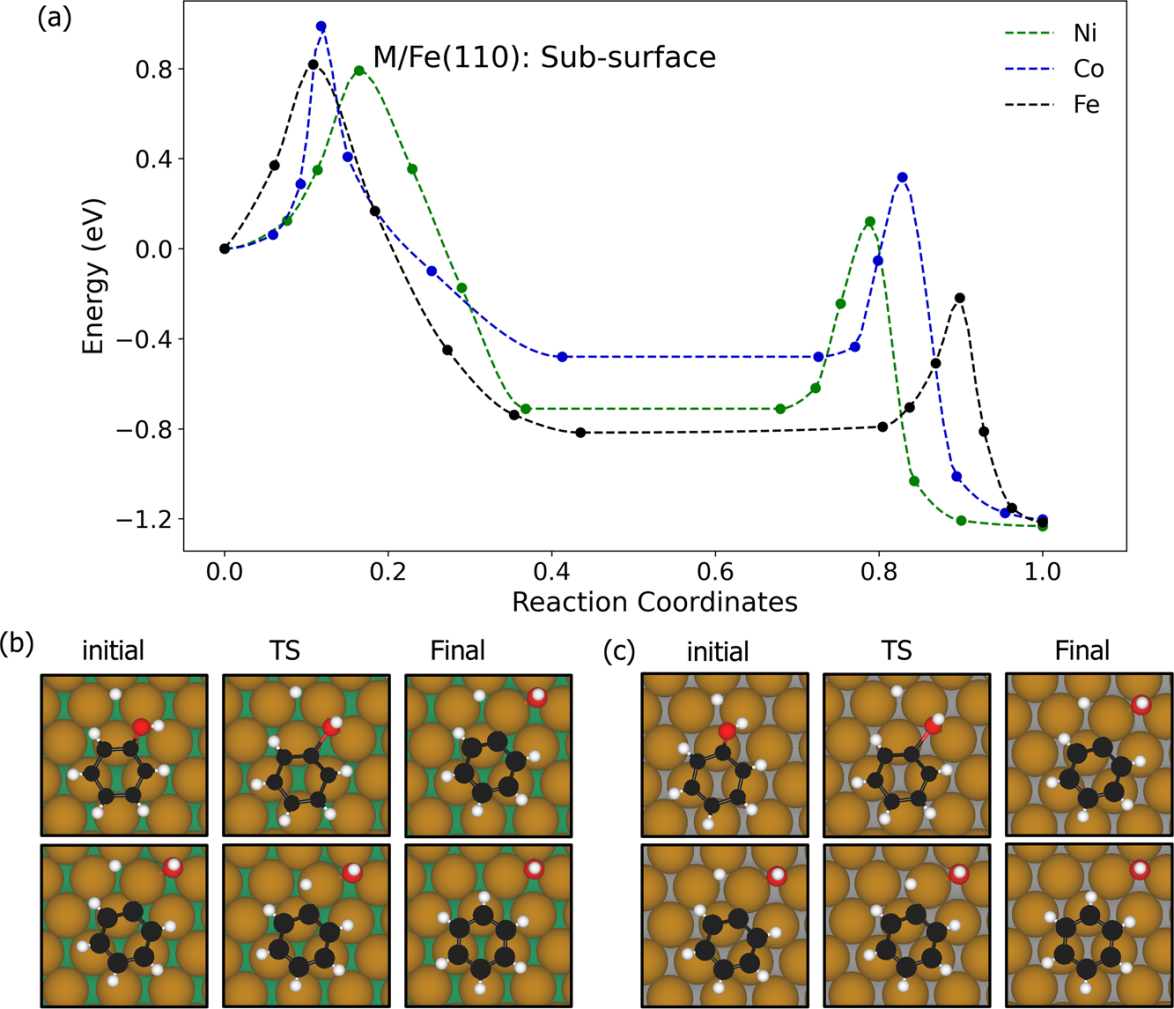
(a) Simulated MEPs of the deoxygenation and hydrogenation steps on the Co and Ni sublayer substitution surfaces. (b) Initial, transition state (TS) and final structures for the C–O bond cleavage (top row) and C–H formation (bottom row) steps on the Ni subsurface. (c) Initial, transition state (TS) and final structures for the same steps for the Co subsurface. Brown, green, gray, white, and red spheres represent Fe, Ni, Co, O, and C atoms, respectively.

The overall reactions are highly exothermic with total energies of −1.20 eV for Co and −1.23 eV for Ni surfaces. Both the C–O bond cleavage and C–H bond formation steps exhibit favorable energetics. The C–O bond cleavage has moderate activation barriers of 0.99 and 0.79 eV with exothermic reaction energies of −0.48 and −0.71 eV for Co and Ni surfaces, respectively. Similarly, the C–H bond formation shows moderate activation barriers of 0.80 and 0.83 eV with exothermic reaction energies of −0.72 and −0.52 eV for Co and Ni surfaces, respectively. The comparable activation barriers between the two steps suggest they compete to be rate-limiting. Overall, the moderate barriers and exothermic nature indicate that DDO is readily facilitated on these sublayer substituted surfaces.

#### Comparisons of Different Surface Models

To further assess the kinetics of the DDO of phenol on these surfaces, we estimated the forward rate constants and equilibrium constants at 350 and 450 °C using the calculated activation and reaction energies, along with the vibrational frequencies of the initial, transition, and final state structures (see Table 1). These temperatures have been shown experimentally to be efficient for HDO of phenolics.^23^

**Table 1:** Calculated Forward Rate Constants (*k*_f_) and Equilibrium Constants (*k*_eq_) for the C–O Cleavage and C–H Formation Reactions at 350 and 450°C

		*T* = 350 °C		*T* = 450 °C	
surface	reaction	*k*_f_ (s^–1^)	*K*_eq_	*k*_f_ (s^–1^)	*K*_eq_
Fe	C–O cleavage	8.9 × 10^6^	1.4 × 10^7^	6.6 × 10^7^	1.6 × 10^6^
	C–H formation	7.3 × 10^8^	6.6 × 10^2^	3.2 × 10^9^	3.0 × 10^2^
Co_top	C–O cleavage	4.9 × 10^2^	7.4 × 10^–2^	2.1 × 10^4^	9.3 × 10^–2^
	C–H formation	8.4 × 10^7^	2.2 × 10^5^	4.7 × 10^8^	4.7*e* × 10^4^
Co_sub	C–O cleavage	1.7 × 10^6^	1.4 × 10^3^	1.6 × 10^7^	4.7 × 10^2^
	C–H formation	2.9 × 10^6^	2.2 × 10^5^	2.0 × 10^7^	4.7 × 10^4^
Ni_top	C–O cleavage	6.1 × 10^–1^	1.3 × 10^–4^	3.6 × 10^1^	3.8 × 10^–4^
	C–H formation	5.2 × 10^7^	4.0 × 10^7^	3.2 × 10^8^	4.2 × 10^6^
Ni_sub	C–O cleavage	9.4 × 10^7^	1.3 × 10^5^	5.2 × 10^8^	2.4 × 10^4^
	C–H formation	5.0 × 10^6^	5.0 × 10^3^	4.3 × 10^7^	1.8 × 10^3^

On the top-layer substitution surfaces (Co_top and Ni_top), the hydrogenation step is thermodynamically favorable. However, the equilibrium constants for the initial C–O cleavage step indicate strong preference for the reverse reaction. On the Ni_top surface the reverse reaction dominates, with *K*_eq_ = 1.3 × 10^–4^ s^–1^ at 350 °C and 3.8 × 10^–4^ s^–1^ at 450 °C, which indicate 3–5 orders of magnitude faster than the forward reaction. On the Co_top surface, the reverse reaction also dominates (*K*_eq_ = 7.4 × 10^–2^ s^–1^ at 350 °C and 9.3 × 10^–2^ s^–1^ at 450 °C), approximately 2–3 orders faster than the forward reaction. This suggests that the C–O cleavage is very unlikely on these surfaces. The unfavorable C–O cleavage kinetics implies that top layer alloying with Ni and Co may not be beneficial for DDO reactions.

On the other hand, the sublayer substitution surfaces (Co_sub and Ni_sub) appears to be a feasible strategy. It offers C–O cleavage kinetics comparable to pure Fe and potentially improving benzene yield through enhanced C–H bond formation. On the Co_sub surface. While C–O cleavage is not enhanced compare to pure Fe, it still proceeds readily. On Co_sub, the forward reaction is about 2–3 orders of magnitude faster (*K*_eq_ = 1.4 × 10^3^ s^–1^ at 350 °C and 4.7 × 10^2^ s^–1^ at 450 °C) than the reverse reaction and approximately 4–5 orders of magnitude fater on Ni_sub (*K*_eq_ = 1.3 × 10^5^ s^–1^ and 2.4 × 10^4^ s^–1^ at the same temperatures). However, the C–H is enhanced on these surfaces. For Co_sub, the forward reaction is 4–5 orders of magnitude faster (*K*_eq_ = 2.2 × 10^5^ s^–1^ at 350 °C and 4.7 × 10^4^ s^–1^ at 450 °C) than the reverse reaction compared to pure Fe (*K*_eq_ = 6.6 × 10^2^ s^–1^ and 3.0 × 10^2^ s^–1^). Ni_sub shows moderate enhancement, with the forward reaction rates of 3–4 orders magnitude faster (*K*_eq_ = 5.0 × 10^3^ s^–1^ and 1.8 × 10^3^ s^–1^).

Hence, Co_sub and Ni_sub surfaces exhibit similar performance to the pure Fe surface. The overall reaction remains highly exothermic, and both elementary steps are thermodynamically and kinetically favorable. This is especially true for the Ni_sub surface, where both the activation barriers and reaction energies are within approximately 0.2 eV of those on the pure Fe surface. While equilibrium constants indicate that C–O cleavage proceeds more readily on the pure Fe surface, the difference is minimal for Ni_sub and more pronounced for Co_sub. These results suggest that subsurface alloying, particularly with Ni, maintains efficient C–O cleavage and substantially improves C–H bond formation, potentially enhancing benzene yield in DDO processes.

## Conclusions

We have demonstrated the effectiveness of the GPR calculator in accelerating NEB calculations for large molecules on surfaces, using phenol on Fe-based bimetallic surfaces as a case study. The GPR calculator significantly reduces the computational cost while maintaining accuracy, achieving up to 3 times speedup compared to pure DFT calculations. Given the high selectivity of Fe-based catalysts for aromatic production and their susceptibility to oxidative deactivation, our results suggest that if Co or Ni are introduced to enhance stability through alloying, subsurface incorporation represents a promising strategy that preserves the catalytic selectivity of Fe-based catalysts. This modification preserves favorable kinetics for C–O bond cleavage and C–H bond formation, comparable to those on pure Fe surfaces. In contrast, top-layer alloying leads to unfavorable C–O cleavage kinetics, indicating it may hinder DDO performance. Overall, these findings underscore the potential of GPR-based methods for efficiently studying complex surface reactions involving larger molecular systems.

## References

[1] Henkelman G., Jónsson H. (2000). Improved tangent estimate in the nudged elastic band
method for finding minimum energy paths and saddle points. J. Chem. Phys..

[2] Henkelman G., Uberuaga B. P., Jónsson H. (2000). A climbing image nudged elastic band
method for finding saddle points and minimum energy paths. J. Chem. Phys..

[3] Herbol H. C., Stevenson J., Clancy P. (2017). Computational Implementation of Nudged
Elastic Band, Rigid Rotation, and Corresponding Force Optimization. J. Chem. Theory Comput..

[4] Jonsson, H. ; Mills, G. ; Jacobsen, K. W. Classical and Quantum Dynamics in Condensed Phase Simulations; World Scientific, 1998; Chapter 16, pp 385–404.

[5] Caspersen K. J., Carter E. A. (2005). Finding transition
states for crystalline solid–solid
phase transformations. Proc. Natl. Acad. Sci.
U.S.A..

[6] Sheppard D., Xiao P., Chemelewski W., Johnson D. D., Henkelman G. (2012). A generalized
solid-state nudged elastic band method. J. Chem.
Phys..

[7] Qian G.-R., Dong X., Zhou X.-F., Tian Y., Oganov A. R., Wang H.-T. (2013). Variable cell nudged elastic band method for studying
solid–solid structural phase transitions. Comput. Phys. Commun..

[8] Peterson A. A. (2016). Acceleration
of saddle-point searches with machine learning. J. Chem. Phys..

[9] Koistinen O.-P., Dagbjartsdottir F. B., Asgeirsson V., Vehtar A., Jonsson H. (2017). Nudged elastic
band calculations accelerated with Gaussian process regression. J. Chem. Phys..

[10] Koistinen O. P., Asgeirsson V., Vehtari A., Jonsson H. (2019). Nudged Elastic Band
Calculations Accelerated with Gaussian Process Regression Based on
Inverse Interatomic Distances. J. Chem. Theory
Comput..

[11] Teng C., Wang Y., Huang D., Martin K., Tristan J. B., Bao J. L. (2022). Dual-Level Training of Gaussian Processes with Physically
Inspired Priors for Geometry Optimizations. J. Chem. Theory Comput..

[12] Teng C., Wang Y., Bao J. L. (2024). Physical Prior Mean
Function-Driven
Gaussian Processes Search for Minimum-Energy Reaction Paths with a
Climbing-Image Nudged Elastic Band: A General Method for Gas-Phase,
Interfacial, and Bulk-Phase Reactions. J. Chem.
Theory Comput..

[13] Schaaf L. L., Fako E., De S., Schäfer A., Csányi G. (2023). Accurate energy barriers for catalytic reaction pathways:
an automatic training protocol for machine learning force fields. npj Comput. Mater..

[14] Onyango I., Kang B., Zhu Q. (2025). GPR_calculator:
An On-the-Fly Surrogate
Model to Accelerate Massive Nudged Elastic Band Calculations. Comput. Phys. Commun..

[15] Nie L., de Souza P. M., Noronha F. B., An W., Sooknoi T., Resasco D. E. (2014). Selective conversion of m-cresol to toluene over bimetallic
Ni–Fe catalysts. J. Mol. Catal. A Chem..

[16] Xiaoyang L., Wei A., Turner C. H., Daniel E. R. (2018). Hydrodeoxygenation of m-cresol over
bimetallic NiFe alloys: Kinetics and thermodynamics insight into reaction
mechanism. J. Catal..

[17] Kordouli E., Kordulis C., Lycourghiotis A., Cole R., Vasudevan P., Pawelec B., Fierro J. (2017). HDO activity
of carbon-supported
Rh, Ni and Mo-Ni catalysts. Mol. Catal..

[18] Huynh T. M., Armbruster U., Pohl M.-M., Schneider M., Radnik J., Hoang D.-L., Phan B. M. Q., Nguyen D. A., Martin A. (2014). Hydrodeoxygenation
of phenol as a model compound for
bio-oil on non-noble bimetallic nickel-based catalysts. ChemCatChem..

[19] Yang F., Liu D., Wang H., Liu X., Han J., Ge Q., Zhu X. (2017). Geometric and electronic effects of bimetallic Ni–Re catalysts
for selective deoxygenation of m-cresol to toluene. J. Catal..

[20] Zhao C., Kasakov S., He J., Lercher J. A. (2012). Comparison
of kinetics,
activity and stability of Ni/HZSM-5 and Ni/Al_2_O_3_-HZSM-5 for phenol hydrodeoxygenation. J. Catal..

[21] Do P. T., Foster A. J., Chen J., Lobo R. F. (2012). Bimetallic effects
in the hydrodeoxygenation of meta-cresol on γ-Al_2_O_3_ supported Pt–Ni and Pt–Co catalysts. Green Chem..

[22] Liu X., An W., Wang Y., Turner C. H., Resasco D. E. (2018). Hydrodeoxygenation
of guaiacol over bimetallic Fe-alloyed (Ni, Pt) surfaces: reaction
mechanism, transition-state scaling relations and descriptor for predicting
C–O bond scission reactivity. Catal.
Sci. Technol..

[23] Sun J., Karim A. M., Zhang H., Kovarik L., Li X. S., Hensley A. J., McEwen J.-S., Wang Y. (2013). Carbon-supported bimetallic
Pd-Fe catalysts for vapor-phase hydrodeoxygenation of guaiacol. J. Catal..

[24] Hong Y., Zhang H., Sun J., Ayman K. M., Hensley A. J., Gu M., Engelhard M. H., McEwen J.-S., Wang Y. (2014). Synergistic Catalysis
between Pd and Fe in Gas Phase Hydrodeoxygenation of m-Cresol. ACS Catal..

[25] Hong Y., Zhang S., Tao F. F., Wang Y. (2017). Stabilization of iron-based
catalysts against oxidation: an in situ ambient-pressure X-ray photoelectron
spectroscopy (AP-XPS) study. ACS Catal..

[26] Nie X., Zhang Z., Wang H., Guo X., Song C. (2021). Effect of
surface structure and Pd doping of Fe catalysts on the selective hydrodeoxygenation
of phenol. Catal. Today.

[27] Zhou J., An W., Wang Z., Jia X. (2019). Hydrodeoxygenation of phenol over
Ni-based bimetallic single-atom surface alloys: mechanism, kinetics
and descriptor. Catal. Sci. Technol..

[28] Li L., Nie X., Chen Y., Janik M. J., Song C., Guo X. (2021). Computational
insights into the hydrodeoxygenation of phenolic compounds over Pt–Fe
catalysts. J. Phys. Chem. C.

[29] Jia X., An W., Wang Z., Zhou J. (2019). Effect of doped metals on hydrodeoxygenation
of phenol over Pt-based bimetallic alloys: Caryl–OH versus
CaliphaticH–OH bond scission. J. Phys.
Chem. C.

[30] Cao L., Mueller T. (2015). Rational design
of Pt3Ni surface structures for the
oxygen reduction reaction. J. Phys. Chem. C.

[31] Wang C., Chi M., Li D., Strmcnik D., Van der Vliet D., Wang G., Komanicky V., Chang K.-C., Paulikas A. P., Tripkovic D. (2011). Design and synthesis of bimetallic electrocatalyst
with multilayered Pt-skin surfaces. J. Am. Chem.
Soc..

[32] Zhifeng J., Weiming W., Zhexi L., Jimin X., Jingguang G. C. (2017). Understanding
the Role of M/Pt(111) (M = Fe, Co, Ni, Cu) Bimetallic Surfaces for
Selective Hydrodeoxygenation of Furfural. ACS
Catal..

[33] Kresse G., Furthmüller J. (1996). Efficiency
of ab-initio total energy calculations for
metals and semiconductors using a plane-wave basis set. Comput. Mater. Sci..

[34] Kresse G., Furthmüller J. (1996). Efficient iterative schemes for ab
initio total-energy
calculations using a plane-wave basis set. Phys.
Rev. B.

[35] Kresse G., Hafner J. (1993). Ab initio molecular
dynamics for liquid metals. Phys. Rev. B.

[36] Kresse G., Joubert D. (1999). From ultrasoft pseudopotentials
to the projector augmented-wave
method. Phys. Rev. B.

[37] Klimeš J. c. v., Bowler D. R., Michaelides A. (2011). Van der Waals
density functionals
applied to solids. Phys. Rev. B.

[38] Grimme S., Hansen A., Brandenburg J. G., Bannwarth C. (2016). Dispersion-corrected
mean-field electronic structure methods. Chem.
Rev..

[39] Hermann J., DiStasio R. A., Tkatchenko A. (2017). First-principles
models for van der Waals interactions in molecules and materials:
Concepts, theory, and applications. Chem. Rev..

[40] Adhikari S., Nepal N. K., Tang H., Ruzsinszky A. (2021). Describing
adsorption of benzene, thiophene, and xenon on coinage metals by using
the Zaremba–Kohn theory-based model. J. Chem. Phys..

[41] Adhikari S., Tang H., Neupane B., Ruzsinszky A., Csonka G. I. (2020). Molecule-surface interaction from
van der Waals-corrected
semilocal density functionals: The example of thiophene on transition-metal
surfaces. Phys. Rev. Mater..

[42] Jia X., An W. (2018). Adsorption of monocyclic
aromatics on transition metal surfaces:
insight into variation of binding strength from first-principles. J. Phys. Chem. C.

[43] Shepard S., Smeu M. (2019). First principles study of graphene
on metals with the SCAN and SCAN+
rVV10 functionals. J. Chem. Phys..

[44] Hensley A. J., Wang Y., McEwen J.-S. (2014). Adsorption of phenol on Fe (110)
and Pd (111) from first principles. Surf. Sci..

[45] Zhu C., Cao J.-P., Yang Z., Zhao X.-Y., Yi W.-C., Feng X.-B., Zhao Y.-P., Bai H.-C. (2022). Study on hydrodeoxygenation
mechanism of anisole over Ni (111) by first-principles calculation. Mol. Catal..

[46] Tran C.-C., Mohan O., Banerjee A., Mushrif S. H., Kaliaguine S. (2020). A combined
experimental and DFT investigation of selective hydrodeoxygenation
of guaiacol over bimetallic carbides. Energy
Fuels.

[47] Methfessel M., Paxton A. T. (1989). High-precision sampling for Brillouin-zone integration
in metals. Phys. Rev. B.

[48] Bitzek E., Koskinen P., Gähler F., Moseler M., Gumbsch P. (2006). Structural
Relaxation Made Simple. Phys. Rev. Lett..

[49] Larsen A. H., Mortensen J. J., Blomqvist J., Castelli I. E., Christensen R., Dułak M., Friis J., Groves M. N., Hammer B., Hargus C. (2017). The atomic simulation environment—a Python library
for working with atoms. J. Phys.: Condens. Matter.

[50] Trygubenko S. A., Wales D. J. (2004). A doubly nudged elastic band method
for finding transition
states. J. Chem. Phys..

[51] Bishop, C. M. ; Nasrabadi, N. M. Pattern Recognition and Machine Learning; Springer, 2006; Vol. 4.

[52] Yanxon H., Zagaceta D., Tang B., Matteson D. S., Zhu Q. (2021). PyXtal_FF:
a python library for automated force field generation. Machine Learning: Sci. Technol..

[53] Zagaceta D., Yanxon H., Zhu Q. (2020). Spectral neural
network potentials
for binary alloys. J. Appl. Phys..

[54] Gershinsky G., Pollak E. (1998). Quantum harmonic transition state theory—Application
to isomerization of stilbene in liquid ethane. J. Chem. Phys..

[55] Laidler, K. Chemical Kinetics; Harper & Row, 1987.

